# Impact of Ki67 re-assessment at time of disease progression in patients with pancreatic neuroendocrine neoplasms

**DOI:** 10.1371/journal.pone.0179445

**Published:** 2017-06-23

**Authors:** Francesco Panzuto, Noemi Cicchese, Stefano Partelli, Maria Rinzivillo, Gabriele Capurso, Elettra Merola, Marco Manzoni, Eugenio Pucci, Elsa Iannicelli, Emanuela Pilozzi, Michele Rossi, Claudio Doglioni, Massimo Falconi, Gianfranco Delle Fave

**Affiliations:** 1 Digestive and Liver Disease Unit, Sant’Andrea Hospital Sapienza University of Rome, Roma, Italy; 2 Pancreatic Surgery Unit, Pancreas Translational & Clinical Research Center, San Raffaele Scientific Institute, “Vita-Salute” University, Milan, Italy; 3 Department of Endocrinology and Internal Medicine, San Raffaele Hospital Scientific Institute, Milan, Italy; 4 Department of Experimental Medicine and Pathology, Sant’Andrea Hospital Sapienza University of Rome, Roma, Italy; 5 Department of Radiology, Sant’Andrea Hospital Sapienza University of Rome, Roma, Italy; 6 Department of Pathology, San Raffaele Scientific Institute, “Vita-Salute” University, Milan, Italy; University of Cordoba, SPAIN

## Abstract

**Background:**

Although re-assessment of proliferative activity by K67 evaluation during the course of neuroendocrine neoplasms (NENs) is recommended in selected patients, its impact on patients’ management is not clear due to the lack of data supporting this practice.

**Aim:**

To investigate Ki67 change at time of progressive disease (PD) in entero-pancreatic NENs (EP-NENs).

**Patients and methods:**

Retrospective analysis of sporadic EP-NENs which received histological re-assessment after PD once radiologically documented.

**Results:**

Forty-three patients were evaluated, including 24 pancreatic NENs (PNENs), and 19 small intestine NENs (SI-NENs). At time of initial histological evaluation, 19 patients had grade 1 (G1) NETs (44.2%), and 24 grade 2 (G2) NETs (55.8%), overall median Ki67 being 3% (range 1%-20%). At time of PD, 13 patients had G1 NETs (30.2%), 26 G2 NETs (60.5%), and 4 had grade 3 (G3) NECs (9.3%), thus resulting in a significant median Ki67 increase (8%, range 1%-70%; p = 0.0006), and a G upgrading in 12 patients (27.9%). A statistically significant Ki67 increase and G grading change at time of PD was observed in PNENs (p = 0.0005 and p = 0.028, respectively). Conversely, no statistically significant change occurred in non-PNENs.

**Conclusions:**

In PNENs with documented PD, Ki67 increase occurs in a significant proportion of patients, providing useful information necessary to choose appropriate therapeutic options.

## Introduction

Entero-Pancreatic Neuroendocrine Neoplasms (EP-NENs) are relatively rare and heterogeneous diseases with different clinical behavior depending on primary tumor site, grading, and staging [1–3].

The most important prognostic factors are primary site, TNM stage, and grading based on tumor proliferative activity, in terms of number of mitosis or proliferative activity, assessed by immunohistochemistry for Ki67. According to the WHO 2010 classification, they are classified into three different categories depending on the Ki67 index: NET G1 (Ki67 ≤ 2%), NET G2 (Ki67: 3–20%), and NEC G3 (Ki67 > 20%) ([4].

Ki67 is considered the strongest predictor for poor clinical outcome in EP—NENs, and it is commonly employed as driver factor to plan therapeutic approaches in these patients.

As EP-NENs often have a long disease course, it is reasonable to hypothesize that their proliferative activity might change over time, and it has been proposed that a histological evaluation to re-assess proliferative activity should be performed in selected cases with rapidly progressive disease, or if imaging information is lacking [5, 6]. The little available data regarding Ki67 changes during the course of the disease suggests that the histological re-assessment in NEN patients may be useful for patients’ management [7, 8]. However, this data is extremely limited and heterogeneous, and despite the above-mentioned recommendations, the real impact of histological re-assessment at time of progressive disease (PD) on patients’ clinical management is not clear.

Thus, the aim of the present study was to investigate Ki67 changes at time of documented PD in patients with advanced EP-NENs.

## Materials and methods

The present study is a retrospective analysis of consecutive patients with sporadic EP-NENs from two Italian centers (Sapienza University of Rome Sant’Andrea Hospital, and Vita-Salute University, San Raffaele Hospital IRCCS, Milan) who, from 2003 to 2016, presented a PD during follow-up with a repeated histological sampling on primary tumor or metastatic lesions in order to re-assess Ki67 at the time of progression.

Histological samplings were taken from surgical specimens or biopsy from primary or metastatic lesions, depending on the clinical scenario. For the purpose of this study, all histological samples (baseline and at time of PD) were re-evaluated by an expert pathologist (E.P. or C.D.) at each center, and classified the tumor according to the WHO 2010 classification [4]. The Ki67 proliferative index was expressed as a percentage based on the count of Ki67-positive cells in areas of the highest immunostaining by manual cell counting, as previously described [9]. According to RECIST criteria 1.0, PD was defined as an increase in lesion number/size or recurrent disease after previous radical surgery, identified by CT scan or MRI [10].

Primary tumors were located in the pancreas or in the small intestine (SI) (in 2 patients, primary tumor origin was unknown, however believed to be of SI origin since primary within the pancreas, chest, or elsewhere was excluded by appropriate diagnostic work-up, and specific immuno-histochemical features supporting SI origin were found).

All patients provided full informed consent before repeating histological assessment.

Values are expressed as median (range). The comparison between subgroups was carried out using Fisher’s exact test, chi-square test, and Mann-Whitney U test or Wilcoxon test, as appropriate, for continuous variables. A p value < 0.05 was considered significant. Statistical analysis was performed using Medcalc (MedCalc Software, http://www.medcalc.org).

## Results

### Histological initial evaluation

A total of 43 patients were included in the present study (S1 Table), including 24 with pancreatic NENs (PNENs) (55.8%), and 19 small intestine NENs (SI-NENs) (44.2%). Thirty-eight patients (88.4%) had non-functioning tumors, whereas the remaining 5 patients (11.6%) had functioning tumor. Patients’ characteristics at time of the first histological evaluation are summarized in Table 1. Histological samples were surgical in 26 cases (60.5%) and bioptic in 17 (39.5%) of patients, and were taken from primary or metastatic lesions in 27 cases (62.8%) and 16 cases (36.2%), respectively. Overall, 19 patients (44.2%) had G1 tumor, whereas the remaining 24 patients (55.8%) had G2 tumor. Median Ki67 was 3% (1%-20%).

**Table 1:** Patients’ general characteristics.

	Overall (43 pts)	PNENs (24 pts)	SI–NENs (19 pts)
**Gender [male; *N* (%)]**	20 (46.5)	10 (41.6)	10 (52.6)
**Median age [years (range)]**	55 (23–74)	49.5 (23–74)	56.5 (45–74)
**Median interval between 1**^**st**^ **and 2**^**nd**^ **histological evaluation [months (range)]**	56 (3–148)	42 (3–148)	58 (5–128)
**Stage IV patients [*N* (%)]**			
**At time of 1**^**st**^ **histological evaluation**	25 (58.1)	12 (50)	13 (68.4)
**At time of 2**^**nd**^ **histological evaluation**	40 (93%)	21 (87.5)	19 (100)

PNENs: pancreatic Neuroendocrine Neoplasms. SI–NENs: small intestine Neuroendocrine Neoplasms

The median interval between the first and the second histological evaluation was 56 months (3–148). Specifically, it was 42 months (3–148) and 61 months (5–128) for PNENs and SI-NENs, respectively (p = 0.385). During this period, 13 patients (30.2%, 8 PNENs and 5 SI-NENs) did not receive medical treatment, since they were disease free after initial radical surgery, whereas the remaining 30 patients (69.8%, 13 PNENs and 17 SI-NENs) were treated by the following therapies: somatostatin analogs (n = 20; 46.5%), PRRT (n = 11; 23.2%), everolimus (n = 7; 16.3%), systemic chemotherapy (n = 4; 9.3%), interferon (n = 3; 7%), liver embolization / ablation (n = 4; 9.3%). Overall, 16 out of 29 patients (55.2%, 10 PNENs and 6 SI-NENs) received two or more therapeutic lines between the two histological assessments.

### Histological evaluation at time of PD

Overall, PD was documented by radiology as an increase in lesions number or size in 30 patients (69.8%, 16 PNENs and 14 SI-NENs), while 13 patients (30.2%, 8 PNENs and 5 SI-NENs) had recurrent disease after previous radical surgery. Histological samples collected at the time of PD were taken from primary tumor in 7 patients (16.3%) and from metastatic lesions in the remaining 36 patients (83.7%). Overall, they were bioptic or surgical specimens in 33 (76.7%) and 10 (23.3%) patients, respectively.

A total of 28 patients (65.1%) showed increased Ki67 at PD in comparison with initial proliferative index assessment. Among these, 20 tumors (71.4%) were PNENs, whereas 8 tumors (28.6%) were SI-NENs. Median Ki67 at time of the second histological evaluation was 8% (range 1–70%) (p = 0.0006 vs median Ki67 at time of initial assessment) (Table 2).

**Table 2:** Tumor grading change at time of progressive disease.

	Overall (n = 43)			PNENs (n = 24)			SI–NENs (n = 19)		
	1^st^ histological evaluation	2^nd^ histological evaluation	P	1^st^ histological evaluation	2^nd^ histological evaluation	P	1^st^ histological evaluation	2^nd^ histological evaluation	P
**G1**	19 (44.2%)	13 (30.2%)	0.074	9 (37.5%)	3 (12.5%)	0.028	10 (52.6%)	10 (52.6%)	0.745
**G2**	24 (55.8%)	26 (60.5%)		15 (62.5%)	17 (70.8%)		9 (47.4%)	9 (47.4%)	
**G3**	-	4 (9.3%)		-	4 (16.7%)		-	-	
**Ki67**	3% (1–20)	8% (1–70)	0.0006	4% (1–15)	11% (1–70)	0.0005	2% (1–20)	2% (1–20)	0.414

PNENs: pancreatic Neuroendocrine Neoplasms. SI–NENs: small intestine Neuroendocrine Neoplasms

Ki 67 is expressed as median (range).

**Fig 1:**
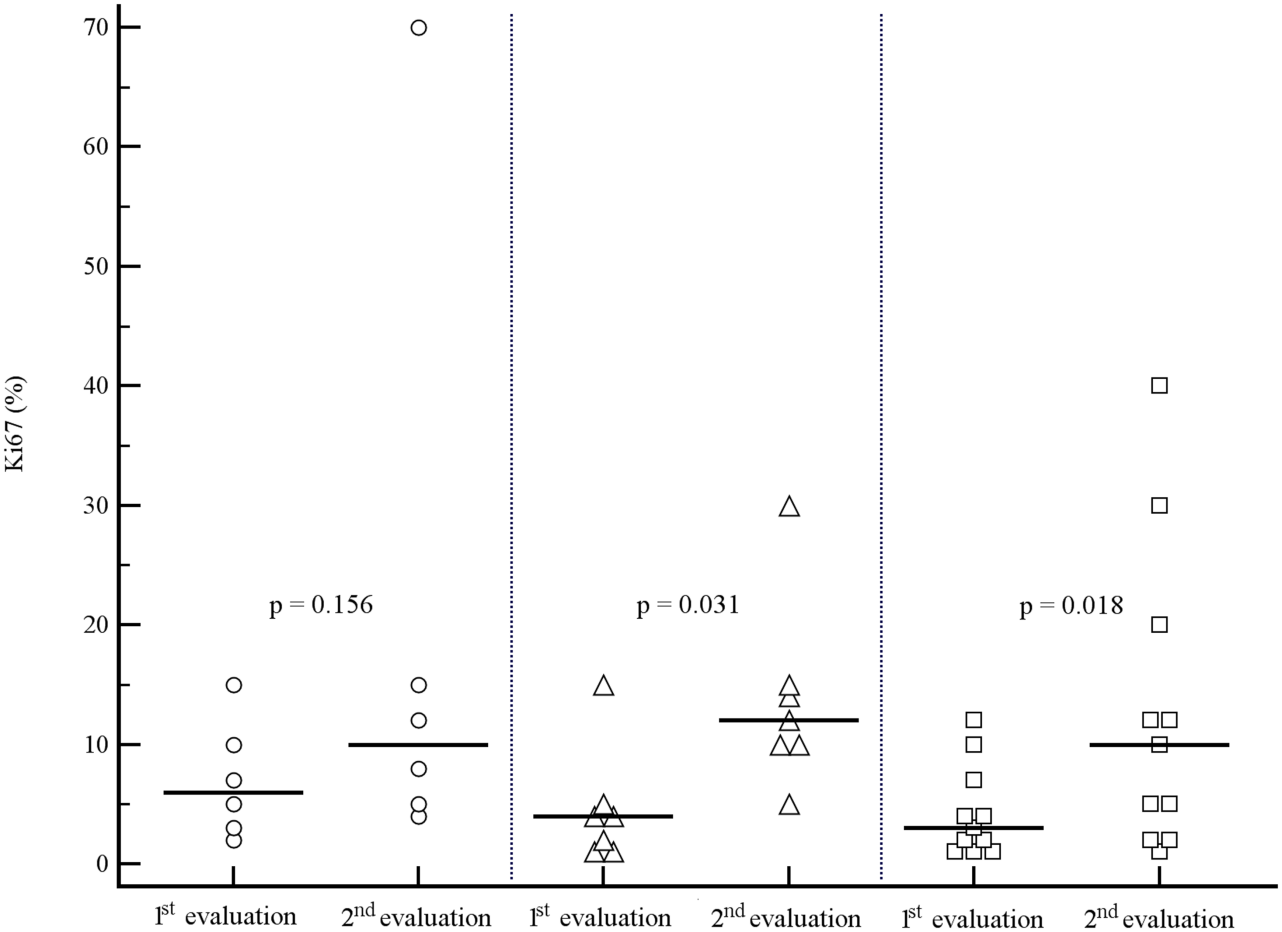
Ki67 change between 1st and 2nd histological evaluation according to the slope of tumor progression. Interval between 1^st^ and 2^nd^ evaluation: < 12 months (circles), 12–48 months (triangles), > 48 months (squares). Horizontal lines = median values.

A trend toward a relationship between an increase in proliferative activity and the length of the interval between initial and repeated histology at time of PD was observed, Ki67 significantly increasing in patients with an interval between the two assessments > 12 months (Fig 1).

**Fig 2:**
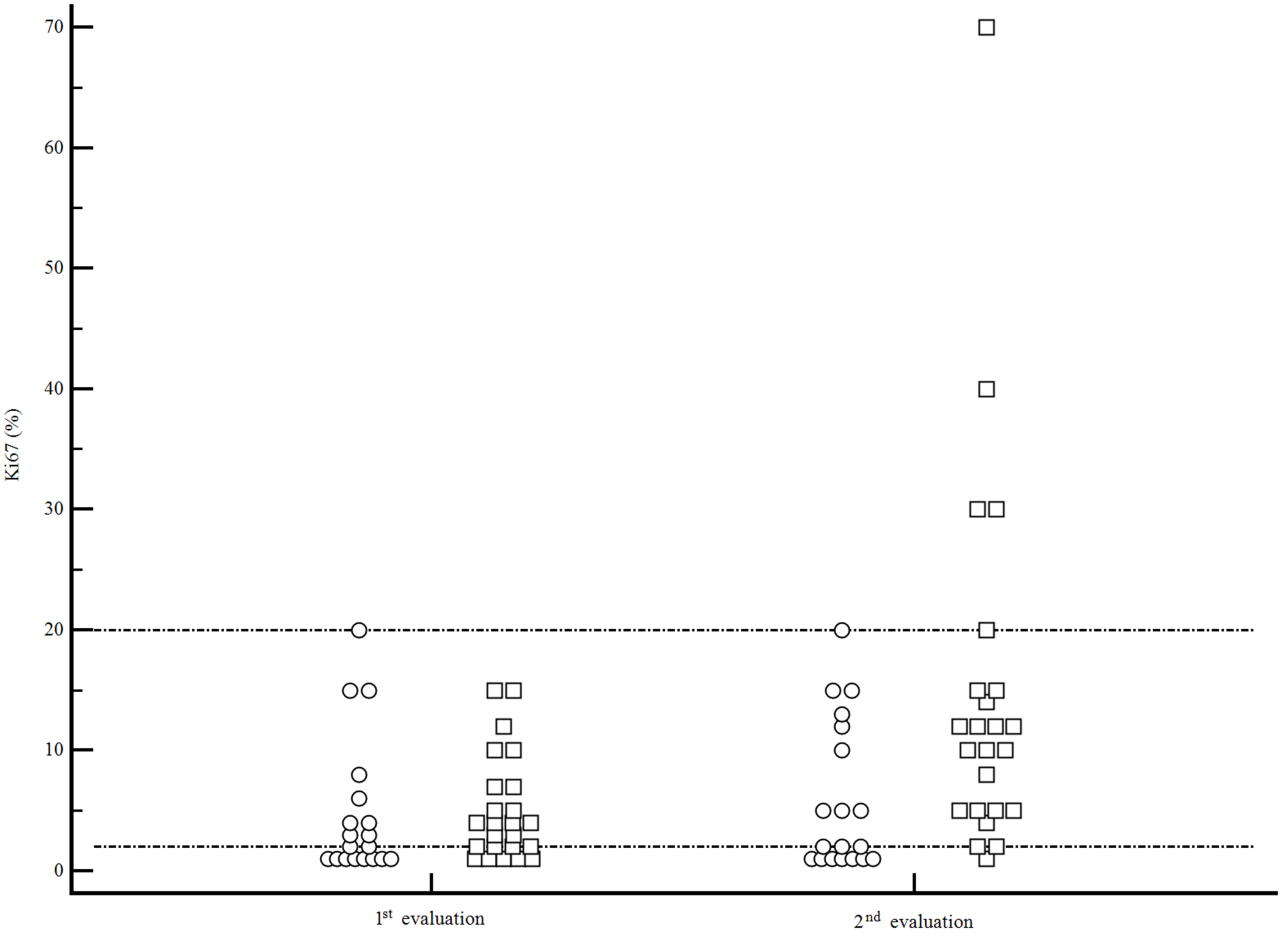
Ki67 change between 1^st^ and 2^nd^ histological evaluation. SI-NENs = circles. PNENs = squares. Horizontal reference lines refer to Ki67 cut-off levels for G1 NENs (2%) and G2 NENs (20%).

Overall, a G grading change was observed in 14 patients (32.6%). Of these, 10 patients had a PNENs (71.4%), and 4 patients had a SI-NENs (28.6%) (Fig 2).

A grading increase was observed in 12 patients (27.9%). Specifically, 8 pts (18.6%) changed from G1 to G2 (6 PNENs, 2 SI-NENs) and 4 patients (9.3%) from G2 to G3 (all PNENs). On the contrary, grading decrease from G2 to G1 occurred in 2 patients (4.6%) with SI-NEN only.

Overall, comparison between grading distribution at the time of first and second histological evaluations was not statistically significant (p = 0.074), however a trend toward an increased proportion of high-grade tumors was observed (Table 2).

As far as the primary tumor site was concerned, proliferative activity significantly changed between the two observations in PNENs with a Ki67 increase from a median value of 4% to 11% (p = 0.0005) (Table 2, Fig 2). In this subset of patients, Ki67 increase occurred after a median period of 42 months (range 3–148 months) from the initial histological assessment. Grading also showed a statistically significant change (p = 0.028) (Table 2). After PD was observed, the therapeutic strategy was changed. Specifically, in the group of patients who reported a G modification, therapeutic changes were as follows: among the group of 8 patients who switched from G1 to G2, 6 patients who were receiving somatostatin analogs (n = 4) or PRRT (n = 2) were treated with systemic chemotherapy (n = 3), everolimus (n = 2), or increased dose of somatostatin analog, respectively (n = 1). The remaining 2 patients were disease free after initial radical surgery, and received systemic chemotherapy after disease recurrence was observed.

The group of 4 patients who switched G grading from G2 to G3 received systemic chemotherapy (n = 2), PRRT (n = 1) or everolimus (n = 1) (these last two patients had a “NET G3”, that is a neuroendocrine carcinoma G3 according to WHO, with a well-differentiated morphology, as previously described [11]).

On the contrary, no significant change in median Ki67 or grading was observed in the subgroup of SI-NENs, in whom similar Ki67 values and grading distribution were observed between initial and repeated histological assessment (p = 0.414 and p = 0.745, respectively) (Table 2, Fig 2).

As far as the pattern of PD is concerned, a significant Ki67 increase at time of PD was observed in patients who reported an increase in size/number of lesions (median 10%, range 1%-40%. P = 0.003 vs initial evaluation), as well as in those who experienced disease recurrence after previous radical surgery (median 5%, range 1%-70%. P = 0.034 vs initial evaluation).

When a subanalysis was performed in the subgroup of 26 patients in whom initial histological evaluation was done on surgical specimens, a significant proliferative activity change was again confirmed, median Ki67 increasing from 3% (range 1%-15%) to 6.5% (range 1%-70%) (p = 0.0009).

## Discussion

The present study shows that an increase in Ki67 rate, as documented by repeating histological assessments, occurs at time of radiologically documented PD in a significant proportion of patients with EP-NENs, particularly in those of pancreatic origin.

Albeit repeating biopsy of metastatic disease to re-assess proliferative activity has been proposed in the literature, especially in the cases with rapidly progressive tumors or if imaging information is lacking [5, 6], very few studies have investigated Ki67 variability over the course of the disease in EP-NEN patients. Thus, due to the lack of scientific data supporting this practice, repeating the histological assessment at time of PD is not currently recommended by guidelines.

The possibility of Ki67 changes throughout the disease course has been reported by Singh et al. in a heterogeneous series of NEN patients including three pancreatic primaries only [7], suggesting that tumor behavior may become more aggressive over time. More recently, an increase in the Ki67 rate was reported to occur in metachronous metastases during follow-up in a small group of 10 patients with EP NENs, including two patients with pancreatic primary tumor only [8].

The use of combined “functional imaging” procedures (i.e. 68Ga-DOTATATE and 18F-FDG PET/CT) has also been proposed as an alternative approach to histological Ki67 evaluation to predict tumor behavior in patients with NENs by Has Simsek et al [12]. However, a small series of 27 patients was evaluated in that study, mainly including tumors with relatively low G grade (Ki67 < 20% in 25 of them), and without information concerning the disease status (stable or progressive) at time of nuclear medicine procedures. To date, proliferative index Ki67 still remains the most reliable factor for therapeutic decision and prognostic assessment in patients with EP-NENs.

In the present study, 65.1% of patients showed an increase in Ki67 value at PD in comparison with initial assessment, thus resulting in a G upgrade in 27.9% of cases. Since grading is considered a key factor to plan management of patients with NENs, Ki67 re-assessment in case of tumor behavior change during follow-up gains a pivotal role for both prognostic evaluation and therapeutic choice. In fact, international guidelines [5, 13] suggest to use somatostatin analogs as first line therapy in G1 and G2 NENs with relatively low Ki67, whereas more aggressive treatments (i.e. targeted therapies, PRRT, or chemotherapy), are proposed with a higher Ki67. In the present study, a change in the therapeutic strategy was chosen in all patients who experienced G grading change after histological re-assessment, confirming that this practice may change clinical management when PD is documented.

Interestingly, the Ki67 change mainly occurred in pancreatic NENs, in which a statistically different G grading distribution was observed at PD in comparison with initial assessment, a figure that was not observed in the non-pancreatic NENs setting (Table 2). This difference may be due to several factors, including the different rate of progression of PNENs in comparison with SI-NENs [14, 15], and the slightly higher proportion of PNENs (8/24 patients, 33.3%) who did not receive treatment between initial and second histological evaluation compared to SI-NENs (5/19 patients, 26.3%). Furthermore, since therapeutic approaches to PNENs and SI-NENs are different, it is not possible to exclude that proliferative activity changes might occur under the effect of the different treatments that patients received between the two histological assessments.

Additional issues need to be taken in account as limitations to the present study, i.e. the retrospective design, which unfortunately is a major inherited pitfall for the majority of the studies on EP-NENs, and the relatively low number of patients evaluated, which is again a common feature of studies evaluating a rare disease like NENs, and that might affect the interpretation of some findings of the present study, including the potential effect of therapy change. Furthermore, a selection bias might exist, as the decision to repeat biopsy was based on the treating physician’s choice. Finally, Ki67 variability between bioptic and surgical specimens, as well as between sites (i.e. primary tumor or metastases) should be considered [7, 16]. Due to these reasons, a larger multicenter study planned with a prospective design is needed to better define the role of histological re-assessment at time of PD in the follow-up algorithms of patients with EP-NENs.

## Conclusions

This study shows that a Ki67 increase occurs in a significant proportion of patients with EP NENs at time of PD, particularly in those with pancreatic origin. This finding suggests that repeating the histological evaluation to re-assess proliferative activity, when PD is documented by radiology, might help planning an appropriate clinical management and therapeutic approach.

## Supporting information

S1 Table Minimal dataset. (XLSX)
